# Validity of the Postoperative Morbidity Survey after abdominal aortic aneurysm repair—a prospective observational study

**DOI:** 10.1186/s13741-015-0020-1

**Published:** 2015-10-12

**Authors:** Ben A. Goodman, Alan M. Batterham, Elke Kothmann, Louise Cawthorn, David Yates, Helen Melsom, Karen Kerr, Gerard R. Danjoux

**Affiliations:** Department of Perioperative Care, Royal Victoria Infirmary, Newcastle upon Tyne, UK; Health and Social Care Institute, Teesside University, Middlesbrough, UK; Department of Academic Anaesthesia, James Cook University Hospital, Marton Road, Middlesbrough, TS4 3BW UK; Department of Anaesthesia, York Hospital, York, UK; Department of Anaesthesia, University Hospital of North Durham, Durham, UK; Department of Anaesthesia, Northern General Hospital, Sheffield, UK

**Keywords:** Aneurysm, AAA, Postoperative, Morbidity, POMS, Complications, Outcome

## Abstract

**Background:**

Currently, there is no standardised tool used to capture morbidity following abdominal aortic aneurysm (AAA) repair. The aim of this prospective observational study was to validate the Postoperative Morbidity Survey (POMS) according to its two guiding principles: to only capture morbidity substantial enough to delay discharge from hospital and to be a rapid, simple screening tool.

**Methods:**

A total of 64 adult patients undergoing elective infrarenal AAA repair participated in the study. Following surgery, the POMS was recorded daily, by trained research staff with the clinical teams blinded, until hospital discharge or death. We modelled the data using Cox regression, accounting for the competing risk of death, with POMS as a binary time-dependent (repeated measures) internal covariate. For each day for each patient, ‘discharged’ (yes/no) was the event, with the elapsed number of days post-surgery as the time variable. We derived the hazard ratio for any POMS morbidity (score 1–9) vs. no morbidity (zero), adjusted for type of repair (endovascular versus open), age and aneurysm size.

**Results:**

The hazard ratio for alive discharge with any POMS-recorded morbidity versus no morbidity was 0.130 (95 % confidence interval 0.070 to 0.243). The median time-to-discharge was 13 days after recording any POMS morbidity vs. 2 days after scoring zero for POMS morbidity. Compliance with POMS completion was 99.5 %.

**Conclusions:**

The POMS is a valid tool for capturing short-term postoperative morbidity following elective infrarenal AAA repair that is substantial enough to delay discharge from hospital. Daily POMS measurement is recommended to fully capture morbidity and allow robust analysis. The survey could be a valuable outcome measure for use in quality improvement programmes and future research.

## Background

The Abdominal Aortic Aneurysm (AAA) Quality Improvement Programme (AAAQIP) was introduced in 2009 with the explicit remit of halving national mortality following elective repair by 2014. Latest published outcomes show the dramatic effect the AAAQIP has had, with mortality figures superior to those initially targeted at 2.4 % (open and endovascular outcomes combined) (Potgieter et al. 2012). As care improves for patients undergoing surgery, mortality becomes less common and therefore becomes a less sensitive measure of differences in the quality of care (Patila et al. 2006). Morbidity following AAA repair is significantly higher than mortality, with an estimated prevalence of 28 % following open repair and 12 % following EVAR (Giles et al. 2010). Despite this, morbidity is not robustly captured in the AAAQIP outcome dataset. Introducing standardised morbidity reporting would represent a logical next step to ensuring transparency of outcomes, whilst providing a more sensitive measure for differences in quality of care.

Capturing postoperative complications is highly relevant given their strong association with adverse outcomes for both patient and institution including prolonged hospital stay, poor functional ability on hospital discharge, reduced life-expectancy, reduced quality of life and increased hospital resource utilisation (Khuri et al. 2005; Head et al. 2008). Despite this, there is no standardised tool used to reliably capture morbidity following AAA repair. Beyond being able to identify complications postoperatively, a robust recording system would also facilitate quality assurance of hospital care and determine the effects of new surgical interventions.

The Postoperative Morbidity Survey (POMS) is a nine-domain tool that prospectively describes in-hospital morbidity following major surgery (Bennett-Guerrero et al. 1999). The POMS is able to detect a range of complication severities—from minor to life threatening—and classify morbidity by organ system, thereby representing a particularly useful scoring system. The original research from which the POMS tool was derived consisted of a heterogeneous population of surgical patients, including a minor cohort undergoing vascular surgery. Subsequent studies demonstrate the POMS has good inter-rater reliability, high patient satisfaction, accurately describe all postoperative complications (Grocott et al. 2007; Davies et al. 2013) and is associated with length of stay (LOS) (Grocott et al. 2007; Davies et al. 2013; Wakeling et al. 2005; Ackland et al. 2007; Ackland et al. 2010; Snowden et al. 2010; Ackland et al. 2011; Sanders et al. 2011; Ausania et al. 2012; Jones et al. 2013; Moonesinghe et al. 2014; West et al. 2014; Lee et al. 2014).

To date, the POMS has not been specifically evaluated in vascular surgery patients. Moreover, the POMS has not been validated against the primary guiding principle for its design—that it should capture morbidity substantial enough to delay discharge from hospital (Bennett-Guerrero et al. 1999). Rather, previous studies have used POMS scores on an arbitrary day post-surgery, for example, day 3 or 5 (Grocott et al. 2007; Davies et al. 2013). The aim of the present study therefore was to validate the POMS following elective AAA repair, both open and endovascular (EVAR), using repeated daily measurement in each patient up to the point of discharge or competing event (death). Consistent with the key guiding principle of the POMS, the primary objective was to estimate the instantaneous relative risk of being discharged from hospital, at any time-point, with any POMS-recorded morbidity (POMS score 1–9) versus no morbidity. A secondary objective was to assess the POMS against the second guiding principle in its development; that it should be a simple and rapid patient-screening tool.

## Methods

This study was a four-centre, prospective observational cohort study that recruited patients from the James Cook University Hospital, Middlesbrough; York Hospital; University Hospital of North Durham and the Northern General Hospital, Sheffield, between July 2012 and March 2013. All patients ≥18 years of age and with capacity to give informed consent, undergoing elective infrarenal AAA repair, were eligible for inclusion. Exclusion criteria were the following: inability to provide informed consent, surgery for suprarenal thoracic or non-elective aneurysms and non-operative management.

A study investigator approached consecutive patients with an information sheet at the preoperative assessment or vascular surgical clinics, and written informed consent was obtained on admission to hospital for the surgical procedure. The study was approved by the West Midlands—Coventry & Warwickshire Research Ethics Committee (reference 12/WM/0114) and locally in recruiting centres (South Tees Hospitals Research and Development Department, York Foundation Trust Research and Development Unit, County Durham and Darlington Centre for Clinical Research and Innovation and the Sheffield Teaching Hospitals Clinical Research Office).

### Study procedure

Patients underwent preoperative assessment and multidisciplinary team discussion based on the AAAQIP care pathway as part of their usual care. Baseline data were collected from preoperative assessment prior to open or endovascular AAA repair. Anaesthetic and surgical techniques were used at the discretion of attending clinicians.

Following surgery, the POMS (Table 1) was recorded daily, by trained research staff, until hospital discharge or death. A modification was made a priori, to reflect changes in clinical practice away from intravenous opioid analgesics towards oral formulations (Pergolizzi et al. 2012). The medical notes were reviewed on a daily basis with any postoperative morbidity not identified by the POMS recorded as free text. Postoperative patient management was otherwise performed per routine clinical practice at the discretion of the attending clinical teams, who were blinded to the POMS data. If patients remained in hospital with no morbidity, the reason for continued admission was recorded as free text. Critical care and overall hospital length of stay were recorded in addition to in-hospital mortality.

**Table 1:** The Postoperative Morbidity Survey (Bennett-Guerrero et al. 1999)

Morbidity type	Criteria	Source of data
Pulmonary	New requirement for supplemental oxygen or other respiratory support	Patient observation
		Treatment chart
Infectious	Currently on antibiotics or temperature >38 °C in the last 24 h	Treatment chart
		Observation chart
Renal	Presence of oliguria (500 mL 24 h^−1^), increased serum creatinine (>30 % from preoperative level) or urinary catheter in place	Patient observation
		Fluid balance chart
		Biochemistry result
Gastrointestinal	Unable to tolerate an enteral diet for any reason, including nausea, vomiting, and abdominal distension *or use of anti-emetic* ^a^	Patient questioning
		Fluid balance chart
		Treatment chart
Cardiovascular	Diagnostic tests or therapy within the last 24 h for any of the following: new myocardial infarction or ischaemia, hypotension (requiring pharmacological therapy or fluid therapy >200 mL h^−1^), atrial or ventricular arrhythmias, cardiogenic pulmonary oedema or thrombotic event (requiring anticoagulation)	Treatment chart
		Note review
Neurological	Presence of new focal deficit, confusion, delirium or coma	Note review
		Patient questioning
Wound	Wound dehiscence requiring surgical exploration or drainage of pus from the operation wound with or without isolation of organisms	Note review
		Pathology result
Haematological	Requirement for any of the following within the last 24 h: packed erythrocytes, platelets, fresh-frozen plasma or cryoprecipitate	Treatment chart
		Fluid balance chart
Pain	New postoperative pain significant enough to require *strong* ^b^ opioids or regional analgesia	Treatment chart
		Patient questioning

^a^Change made by Grocott et al. 2007

^b^Change for the present study (previously *parenteral*)

### Sample size and statistical analysis

Sample size and power estimations were conducted using R® software (Qiu et al. 2012). We predicted that at least 60 patients could be recruited over 9 months. This sample size provides 85 % power to detect a clinically relevant hazard ratio of ~0.4 reflecting a substantially lower instantaneous relative risk of being discharged at any time with any POMS-recorded morbidity versus no morbidity (with 2*P* = 0.05, an expected discharge rate of 95 %, a predicted proportion of the sample with POMS morbidity at discharge of 25 % and a correlation coefficient of 0.1 between the primary exposure (POMS) and the other covariates in the model (see below).)

We analysed the data using a simple multistate Cox regression model to derive the cause-specific association between morbidity and hospital discharge (alive), accounting for the competing risk of in-hospital death (Beyersmann et al. 2012). For each day for each patient, ‘discharged’ (yes/no) was the event, with the elapsed number of days post-surgery as the time variable. The POMS score (yes = 1–9, no = zero) was a binary time-dependent internal covariate. Analysis was conducted using Stata® software (StataCorp. 2013. *Stata Statistical Software: Release 13*. College Station, TX: StataCorp LP). The hazard ratio (95 % confidence interval) for the effect of any POMS morbidity vs. no morbidity was estimated, controlling for type of repair (open vs. EVAR), patient age and size of aneurysm. These three variables were selected, a priori, as each may be predicted to affect length of stay independent of POMS morbidity (Schouten et al. 2006; Lee et al. 2004). Sex was not included as a covariate as the sample was 95 % male. The hazard ratio reflects the instantaneous relative risk of alive discharge for a state of morbidity vs. no morbidity.

One patient was discharged 8 days post-surgery but due to administrative error had no POMS scores recorded after day 5. To include this patient’s event data in the analysis, we carried forward the day 5 morbidity (in the gastrointestinal domain) for days 6–8—a conservative approach in this context.

With respect to the validity of POMS as conceptualised in the current study, there are two types of ‘error’ versus the reference method of the discharge decision by the vascular team. First, a patient might attain a zero score for POMS on a particular day but not be discharged within 24 h of that time-point. Second, a patient might be discharged despite having recorded POMS morbidity. We present a summary of both of these categories in the ‘Results’ section.

We also conducted a sensitivity analysis to examine the effect that patients who were not discharged within 24 h of achieving a zero score had on the validity of the POMS (degree of attenuation of the observed hazard ratio). To conduct this analysis, we first manually edited the data to reflect a scenario in which all patients who reached a zero for POMS had been discharged immediately (i.e. within 24 h).

## Results

### Participant flow

Out of the 104 potential participants screened for inclusion, 81 met the inclusion criteria and were approached by an investigator, of which 64 were recruited into the study. Reasons for non-enrolment were non-operative management (*n* = 8), surgery performed outside recruitment window (*n* = 5), refusal (*n* = 3) and cognitive dysfunction precluding informed consent (*n* = 1). All 64 patients completed follow-up and were included in the analysis.

### Sample characteristics

Patient and perioperative characteristics are described in Table 2. Prevalence of daily presence of POMS morbidity and the count of the number of domains affected are presented in Table 3. Data are presented up to day 6, when approximately 50 % of the overall sample had been discharged. One patient (1.6 % of the total sample) died on the ninth postoperative day after an open AAA repair from multi-organ failure. One patient had postoperative limb ischaemia suspected clinically, resulting in further investigation, but did not meet any of the existing POMS domain criteria.

**Table 2:** Patient and perioperative characteristics

Variable	*n*	Total (*n* = 64)	EVAR (*n* = 31)	Open (*n* = 33)
Age (years)	64	72.7 (7.9)	75.6 (6.2)	70.0 (8.5)
Male:female	64	61:3	29:2	32:1
Body mass index (kg m^−2^)	62	28.2 (5.0)	28.0 (4.6)	28.4 (5.4)
ASA-PS	64			
2		11	1	10
3		47	26	21
4		6	4	2
Revised cardiac risk index (Lee et al. 1999)	64			
1		26	12	14
2		30	16	14
3		8	3	5
Estimated glomerular filtration rate (Levey et al. 1999) (ml min^−1^)	64	76.7 (21.5)	75.9 (22.8)	77.6 (20.6)
Size of AAA (cm)	64	6.7 (1.3)	6.4 (1.2)	7.1 (1.4)
Duration of procedure (h)	57	3.2 [2.2–5.4]	2.5 [2.0–3.6]	4.0 [3.0–6.3]
Aortic cross-clamp time (h)	28	1.1 [0.8–1.8]	n/a	1.1 [0.9–1.8]
Estimated blood loss (L)	44	1.2 [0.5–3.8]	0.5 [0.3–0.6]	2 [1.2–4.6]
Type of anaesthetic	64			
General		45	12	33
Spinal		4	4	0
Epidural		32	2	30
Combined spinal-epidural		14	14	0
In-hospital mortality	64	1	0	1
Postoperative LOS (days)	64	6 [4–11]	4 [3–6]	9 [6–13]
Critical care LOS (days)	64	2 [1–4]	1 [1–2]	4 [2–6]

Data are mean (SD), median [interquartile range] or number

*ASA-PS* American Society of Anesthesiologists Physical Status, *LOS* length of stay, *EVAR* endovascular aneurysm repair

**Table 3:** Prevalence of any POMS-recorded morbidity (yes/no) and POMS count for those patients remaining in hospital at that time-point

	EVAR (*n* = 31)		Open (*n* = 33)		All (*n* = 64)	
Day	Prevalence	Count	Prevalence	Count	Prevalence	Count
1	100 %	3 (2–3)	100 %	5 (4–6)	100 %	4 (3–5)
2	74 %	1 (0–3)	100 %	4 (4–5)	88 %	3.5 (1–4)
3	39 %	0.5 (0–2)	100 %	4 (2–5)	70 %	2 (1–4)
4	29 %	0 (0–2)	85 %	3 (1–5)	58 %	2 (0–4)
5	26 %	1 (0–1)	76 %	2.5 (1–4.75)	52 %	1 (0–4)
6	19 %	1 (0.25–1)	70 %	2 (0.5–5)	45 %	1 (0.5–4)

Data are % of total *n*, median (interquartile range)

*EVAR* endovascular aneurysm repair

### Validity of the POMS

Compliance with POMS completion was 99.5 %, with just three missing days for one patient from a total of 608 possible observations (see ‘Methods’). The hazard ratio for discharge with any POMS-recorded morbidity versus no morbidity was 0.130 (95 % confidence interval 0.070 to 0.243). The median time-to-discharge was 13 days after recording any POMS morbidity vs. 2 days after scoring zero for POMS morbidity.

Twenty-nine patients remained in hospital beyond 24 h of first attaining a zero score for POMS. For 10 of these patients, nothing was documented in the patient notes that might shed light on reasons for any delay. For the other 19 patients, the notes suggested that 4 were awaiting the results of diagnostic tests, 7 had evidence of social/non-medical reasons that delayed discharge, 3 remained in hospital for medical reasons, and 5 developed morbidity very shortly after first reaching zero POMS morbidity: 2 with an infective complication, 2 with pain, and 1 with atrial fibrillation. The sensitivity analysis assuming that all of these 29 patients had been discharged when they first reached a zero score for POMS revealed a hazard ratio for morbidity vs. no morbidity of 0.058 (0.030 to 0.114).

Nineteen patients were discharged with a POMS score of >0 (morbidity in one or more POMS domains). The majority of these (*n* = 17) had recorded morbidity in a single POMS domain: 10 for Infectious, 4 for Pain, and one each for Gastrointestinal, Cardiovascular and Pulmonary. Two patients had recorded morbidity in two POMS domains—Cardiovascular plus Pain and Infectious plus Pain.

## Discussion

### Validity

This is the first study to validate the POMS specifically in patients undergoing abdominal aortic aneurysm repair. The observed hazard ratio reveals that the chances of being discharged by usual clinical decision-making at any time-point with any POMS-recorded morbidity are approximately 1/8 those of being discharged in the absence of POMS morbidity—a very large effect size. There was also a very large difference for the median time-to-discharge in the morbidity vs. no morbidity states. Taken together, these findings provide strong evidence for the validity of the POMS against its primary guiding principle—to only capture morbidity that is of a type and severity to delay discharge from hospital. Following abdominal aortic aneurysm repair the POMS therefore has good, albeit not perfect, validity.

The factor primarily responsible for the non-perfect validity of the POMS in this context is the 19 patients who were discharged with a POMS score >0. The majority of these patients had POMS-recorded morbidity in a single domain—most frequently ‘Infectious’, followed by ‘Pain’. If these 19 patients had instead not been discharged (censored data, i.e. ‘event’ did not occur) then the hazard ratio for morbidity vs. no morbidity would have been essentially zero, as the POMS morbidity covariate at the point of discharge would have been constant across the sample. The sensitivity analysis revealed that the 29 patients who were not discharged within 24 h of first reaching a POMS score of zero attenuated the observed hazard ratio by >50 % (0.130 vs. 0.058). This implies that if all 29 had in fact been discharged within 24 h of first reaching zero, the chances of being discharged with a POMS score >0 compared to a POMS score =0 would decrease significantly from 1/8 (the value we observed) to 1/17. This difference can be explained by imperfections in the healthcare system (delayed recognition of recovery from perioperative morbidity) or by the presence of factors preventing discharge that were not identified by either the POMS or the patient notes—see below for a critique of the POMS.

With respect to delayed discharge, hospital length of stay is affected by social and organisational factors unrelated to the patients’ physiological recovery from surgery (Bennett-Guerrero et al. 1999; Grocott et al. 2007). Grocott et al. reported that 161 out of 200 patients (81 %) remained in hospital with no identifiable morbidity following major surgery (Grocott et al. 2007). In contrast, the current study found fewer than half of the patients stayed in hospital beyond the day on which POMS first identified zero morbidity. This disparity could be due to differences between the study samples, differing discharge practices between institutions (de Jong et al. 2006) or the emphasis on throughput and efficiency in the NHS in recent years (Paton et al. 2014), resulting in more prompt discharge once postoperative morbidity has resolved.

### Potential use

Our strategy of collecting daily POMS count facilitates objective ‘morbidity tracking’ and discharge planning for individual patients in the postoperative period. Considering the former, progressive improvements in POMS count can be used as an indicator of patient recovery leading towards appropriately targeted discharge planning once no morbidity is recorded. Conversely, an upward trend in POMS count should trigger appropriate senior level review and reconsideration of ‘fitness for discharge’. In this respect, we propose POMS to be an adjunct, rather than replacement, for other regularly recorded physiological scoring systems, e.g. National Early Warning Score (NEWS) (Royal College of Physicians of London 2012). The organ-specific structure of the POMS may guide clinicians making diagnostic and treatment decisions in the postoperative period, over and above the non-specific information from NEWS. Using two complementary systems enables early identification of postoperative complications, when patients can be more easily rescued. This could be achieved without undue time-burden on attending healthcare professionals as we expand on below.

The concept of enhanced discharge decision-making is particularly pertinent when considering the aforementioned pressures to increase efficiency and decrease hospital length of stay. This situation creates a significant risk of discharging patients before they are truly ready, resulting in increased readmissions (Kaboli et al. 2012) and resultant financial penalties for NHS trusts (Department of Health 2013). The objective nature of POMS provides a safeguard against this particularly with daily tracking. Standardising the level of recovery at which patients are discharged through use of the POMS could therefore prevent both premature and late discharge. In addition, the objective nature enables nurse initiated discharge and reduction in burden on senior medical staff.

Contrary to widespread belief, we believe that it is indeed permissible to sum POMS domains into a total score. The misconception is based on the low–moderate internal consistency reported for the nine POMS domains, which has led researchers into believing that the items may not be summed into a unidimensional scale (Grocott et al. 2007; Davies et al. 2013; Moonesinghe et al. 2014). However, the POMS is clearly composed of so-called ‘causal indicators’ rather than ‘effect indicators’ (Streiner 2003). With *scales* composed of effect indicators, the items are manifestations of an underlying construct. For example, in a scale consisting of multiple items to measure the construct of depression, scores for all items would be expected to increase or decrease together if depression got worse or better, respectively. For such scales, therefore, unidimensionality (evaluated via confirmatory factor analysis) and high internal consistency are indeed important. However, for *indexes* composed of causal indicators, where the items taken together define the construct—in this case postoperative morbidity—internal consistency is not relevant. Indeed, the low–moderate correlation between the POMS domains is a direct consequence of its design, focusing on multiple physiological systems. In short, these domains are sometimes largely independent (e.g. ‘Pain’ and ‘Renal’) and other times correlated (e.g. ‘Wound’ and ‘Infectious’). This differentiation between a scale and an index is critical; with due caution, the nine domains of the POMS may be summed into an index to reflect the magnitude of postoperative morbidity. This index should be validated in larger studies prior to further use.

### Critique of the POMS

A guiding principle in the development of the POMS was that data collection should be low-burden. Feedback from the research team confirmed this to be true and compliance with POMS completion was excellent in the current study. Indeed, the simplicity of the POMS is one of its core strengths. It has previously been shown to have good reliability even when used by researchers previously unfamiliar with its use (Davies et al. 2013). With definitions that are understandable and simple to answer by the clinical team caring for patients, it could easily be incorporated into routine postoperative monitoring. We chose to keep the existing structure of POMS to enable this goal. In comparison, the recently developed and validated cardiac-POMS (Sanders et al. 2012) changed and added definitions to develop a 13-domain survey suited to patients recovering from cardiac surgery. However, our data do suggest one change to the POMS to make it suitable for patients undergoing vascular surgery: the addition of *limb ischaemia* to the cardiovascular domain definition.

The POMS may not capture all relevant reasons why patients remain in hospital following surgery. General deconditioning and reduced mobility can require a period of rehabilitation, delaying hospital discharge without falling into one of the POMS domains. In the study by Grocott et al. (Grocott et al. 2007), 41 out of 200 patients remained in hospital on day 8 due to postoperative mobility problems. No formal assessment of reduced mobility was used in that study or in ours. Had one been used, it may have revealed some of the unexplained delayed discharges. The newly developed cardiac-POMS added a separate domain for assisted ambulation (Sanders et al. 2012). Impaired mobility can arguably be considered as postoperative morbidity that is currently unrecognised by the POMS and could be addressed in future research with a similar modification. However, any such change would have to be carefully evaluated, as adding further domains would increase the complexity of the tool, making it more burdensome to use.

Several alternative instruments have been developed to measure morbidity following surgery, although many have not gained widespread popularity (Clavien et al. 1992; Pomposelli et al. 1997; Gawande et al. 1999; Pillai et al. 1999; Dindo et al. 2004; Slankamenac et al. 2013; Strasberg et al. 2009). The Clavien-Dindo classification of postoperative complications (Dindo et al. 2004) is perhaps the most commonly used alternative system. It retrospectively classifies complications by type of treatment required to manage them. Some of the problems encountered with the system include as follows difficulties in categorising some cases (Clavien et al. 2009), differing morbidity may be classified similarly and the same complication may be classified differently depending on the treatment given (Rassweiler et al. 2012)—although this criticism can also be levelled at the POMS. It is unclear whether the original description and validation study of the Clavien-Dindo classification (Dindo et al. 2004) or its subsequent analysis (Clavien et al. 2009) specifically assessed its use in vascular surgery. Although it has subsequently been used in several publications in the vascular literature (Pol et al. 2011; Desai et al. 2011; Arya et al. 2015; Visser et al. 2014; Patel et al. 2015; Gunawansa et al. 2011), it was not used by the AAAQIP (AAAQIP 2011). The National Surgical Quality Improvement Program has been widely adopted in the USA, but the definitions of the postoperative complications are not publically available (https://www.facs.org/quality-programs/acs-nsqip). A high-profile pilot scheme is soon to be launched across the UK in a combined venture between the Health Services Research Centre and National Institute of Academic Anaesthesia. The Perioperative Quality Improvement Programme aims to capture postoperative complications, mortality and patient reported outcome in a structured fashion utilising using a range of validated instruments (Moonesinghe and Grocott 2015). This venture should provide further much needed clarity around the optimal strategy for recording postoperative complications.

### Strengths and limitations

The key strength of the current study is that it is the first to validate the POMS according to its primary guiding design principle—to only capture morbidity substantial enough to delay discharge from hospital. This required careful blinding of the clinical teams to the POMS scores and daily POMS recording up until the point of discharge or competing event (a range of over 3 months). A limitation is the relatively small sample. However, this sample size, together with multiple measures per patient, afforded adequate precision to estimate the effect of any morbidity versus no morbidity. Our design is statistically very efficient, as the expected event rate (discharge) is very high (>95 %) which results in a smaller sample size requirement versus studies in which the outcome occurs less frequently. Moreover, our sample is representative of this patient population, with age and length of stay for EVAR and open repair patients equivalent to national data (Potgieter et al. 2012). Furthermore, the multi-centre design of our study adds to its external validity. The institutions include those traditionally described as both teaching hospitals and district general hospitals, reflecting current practice in the UK.

Another limitation is the lack of observation following hospital discharge. Nineteen patients were discharged with POMS morbidity recorded. Given the experience of previous researchers, where patients remained in hospital with no postoperative morbidity (Grocott et al. 2007), we did not consider the alternative situation when planning the study. It may have been informative to have followed these participants after hospital discharge to see whether they had an increased rate of re-admission or use of primary care.

From a wider-reaching perspective, robust postoperative morbidity capture is highly relevant. The development of early postoperative complications is of greater prognostic significance than preoperative comorbidity in determining long-term survival (Khuri et al. 2005). In this setting, although the POMS describes only in-hospital morbidity, a recent study demonstrated its association with increased mortality for up to 2 years following surgery (Moonesinghe et al. 2014). In addition, objective postoperative morbidity data would add to the existing mortality and length of stay information used to benchmark institutions comparatively. Furthermore, daily POMS recording as part of perioperative care pathways might highlight areas for improvement and be used to audit any resultant change in practice. The POMS could therefore act as an informative tool both locally and nationally to drive quality improvement and institution benchmarking.

## Conclusions

This study has demonstrated the POMS to be a valid tool to measure short-term postoperative morbidity in patients undergoing elective infrarenal AAA repair. The data suggest a minor modification to ensure complete capture of morbidity. Daily POMS measurement is recommended to fully capture morbidity and allow robust analysis. The survey could be a valuable outcome measure for use in quality improvement programmes and future research, or as an adjunct to clinical decision-making in the postoperative period.

## Abbreviations

AAA: abdominal aortic aneurysm
AAAQIP: National Abdominal Aortic Aneurysm Quality Improvement Programme
EVAR: endovascular aneurysm repair
LOS: length of stay
NHS: National Health Service
POMS: Postoperative Morbidity Survey

## References

[CR1] AAAQIP (2011). National Abdominal Aortic Aneurysm Quality Improvement Programme Interim report.

[CR2] Ackland GL, Scollay JM, Parks RW, De Beaux I, Mythen MG (2007). Pre‐operative high sensitivity C‐reactive protein and postoperative outcome in patients undergoing elective orthopaedic surgery. Anaesthesia.

[CR3] Ackland GL, Harris S, Ziabari Y, Grocott M, Mythen M (2010). Revised cardiac risk index and postoperative morbidity after elective orthopaedic surgery: a prospective cohort study. Br J Anaesth.

[CR4] Ackland GL, Moran N, Cone S, Grocott MPW, Mythen MG (2011). Chronic kidney disease and postoperative morbidity after elective orthopedic surgery. Anesth Analg.

[CR5] Arya S, Kim SI, Duwayri Y, Brewster LP, Veeraswamy R, Salam A, Dodson TF (2015). Frailty increases the risk of 30-day mortality, morbidity, and failure to rescue after elective abdominal aortic aneurysm repair independent of age and comorbidities. J Vasc Surg.

[CR6] Ausania F, Snowden CP, Prentis JM, Holmes LR, Jaques BC, White SA, French JJ, Manas DM, Charnley RM (2012). Effects of low cardiopulmonary reserve on pancreatic leak following pancreaticoduodenectomy. Br J Surg.

[CR7] Bennett-Guerrero E, Welsby I, Dunn TJ, Young LR, Wahl TA, Diers TL, Phillips-Bute BG, Newman MF, Mythen MG (1999). The use of a postoperative morbidity survey to evaluate patients with prolonged hospitalization after routine, moderate-risk, elective surgery. Anesth Analg.

[CR8] Beyersmann J, Schumacher M, Allignol A. Time-dependent covariates and multistate models. In: Competing Risks and Multistate Models with R. New York: Springer; 2012. p. 211–26. [Use R!].

[CR9] Clavien PA, Sanabria JR, Strasberg SM (1992). Proposed classification of complications of surgery with examples of utility in cholecystectomy. Surgery.

[CR10] Clavien PA, Barkun J, de Oliveira ML, Vauthey JN, Dindo D, Schulick RD, de Santibañes E, Pekolj J, Slankamenac K, Bassi C, Graf R, Vonlanthen R, Padbury R, Cameron JL, Makuuchi M (2009). The Clavien-Dindo classification of surgical complications: five-year experience. Ann Surg.

[CR11] Davies SJ, Francis J, Dilley J, Wilson RJT, Howell SJ, Allgar V (2013). Measuring outcomes after major abdominal surgery during hospitalization: reliability and validity of the Postoperative Morbidity Survey. Perioper Med.

[CR12] de Jong JD, Westert GP, Lagoe R, Groenewegen PP (2006). Variation in hospital length of stay: do physicians adapt their length of stay decisions to what is usual in the hospital where they work?. Health Serv Res.

[CR13] Department of Health. Payment by Results Guidance for 2013–14. 2013.

[CR14] Desai M, Gurusamy KS, Ghanbari H, Hamilton G, Seifalian AM. Remote ischaemic preconditioning versus no remote ischaemic preconditioning for vascular and endovascular surgical procedures. In: Cochrane Database of Systematic Reviews. John Wiley & Sons, Ltd; 2011. 10.1002/14651858.CD008472.pub2 22161429

[CR15] Dindo D, Demartines N, Clavien P-A (2004). Classification of surgical complications. Ann Surg.

[CR16] Gawande AA, Thomas EJ, Zinner MJ, Brennan TA (1999). The incidence and nature of surgical adverse events in Colorado and Utah in 1992. Surgery.

[CR17] Giles KA, Wyers MC, Pomposelli FB, Hamdan AD, Avery Ching Y, Schermerhorn ML (2010). The impact of body mass index on perioperative outcomes of open and endovascular abdominal aortic aneurysm repair from the National Surgical Quality Improvement Program, 2005–2007. J Vasc Surg.

[CR18] Grocott MPW, Browne JP, Van der Meulen J, Matejowsky C, Mutch M, Hamilton MA, Levett DZH, Emberton M, Haddad FS, Mythen MG (2007). The Postoperative Morbidity Survey was validated and used to describe morbidity after major surgery. J Clin Epidemiol.

[CR19] Gunawansa N, Goonerathne T, Cassim R, Wijeyaratne M (2011). Open repair of infra renal abdominal aortic aneurysms: a single center experience from the developing world. Ann Vasc Dis.

[CR20] Head J, Ferrie JE, Alexanderson K, Westerlund H, Vahtera J, Kivimäki M (2008). Diagnosis-specific sickness absence as a predictor of mortality: the Whitehall II prospective cohort study. BMJ.

[CR21] Jones C, Kelliher L, Dickinson M, Riga A, Worthington T, Scott MJ, Vandrevala T, Fry CH, Karanjia N, Quiney N (2013). Randomized clinical trial on enhanced recovery versus standard care following open liver resection. Br J Surg.

[CR22] Kaboli PJ, Go JT, Hockenberry J, Glasgow JM, Johnson SR, Rosenthal GE, Jones MP, Vaughan-Sarrazin M (2012). Associations between reduced hospital length of stay and 30-day readmission rate and mortality: 14-year experience in 129 Veterans Affairs Hospitals. Ann Intern Med.

[CR23] Khuri SF, Henderson WG, DePalma RG, Mosca C, Healey NA, Kumbhani DJ (2005). Determinants of long-term survival after major surgery and the adverse effect of postoperative complications. Ann Surg.

[CR24] Lee TH, Marcantonio ER, Mangione CM, Thomas EJ, Polanczyk CA, Cook EF, Sugarbaker DJ, Donaldson MC, Poss R, Ho KKL, Ludwig LE, Pedan A, Goldman L (1999). Derivation and prospective validation of a simple index for prediction of cardiac risk of major noncardiac surgery. Circulation.

[CR25] Lee WA, Carter JW, Upchurch G, Seeger JM, Huber TS (2004). Perioperative outcomes after open and endovascular repair of intact abdominal aortic aneurysms in the United States during 2001. J Vasc Surg.

[CR26] Lee A, Chiu CH, Cho MWA, Gomersall CD, Lee KF, Cheung YS, Lai PBS (2014). Factors associated with failure of enhanced recovery protocol in patients undergoing major hepatobiliary and pancreatic surgery: a retrospective cohort study. BMJ Open.

[CR27] Levey AS, Bosch JP, Lewis JB, Greene T, Rogers N, Roth D (1999). A more accurate method to estimate glomerular filtration rate from serum creatinine: a new prediction equation. Ann Intern Med.

[CR28] Moonesinghe R, Grocott M (2015). Towards a national Perioperative Quality Improvement Programme (PQIP). Bull R Coll Anaesth.

[CR29] Moonesinghe SR, Harris S, Mythen MG, Rowan KM, Haddad FS, Emberton M, Grocott MPW (2014). Survival after postoperative morbidity: a longitudinal observational cohort study. Br J Anaesth.

[CR30] National Surgical Quality Improvement Program. [https://www.facs.org/quality-programs/acs-nsqip].

[CR31] Patel SD, Constantinou J, Simring D, Ramirez M, Agu O, Hamilton H, et al. Results of complex aortic stent grafting of abdominal aortic aneurysms stratified according to the proximal landing zone using the Society for Vascular Surgery classification. J Vasc Surg. 2015;62:319–25.e2. 10.1016/j.jvs.2015.03.035 25943455

[CR32] Patila T, Kukkonen S, Vento A, Pettila V, Suojaranta-Ylinen R (2006). Relation of the sequential organ failure assessment score to morbidity and mortality after cardiac surgery. Ann Thorac Surg.

[CR33] Paton F, Chambers D, Wilson P, Eastwood A, Craig D, Fox D, et al. Initiatives to reduce length of stay in acute hospital settings: a rapid synthesis of evidence relating to enhanced recovery programmes. Health Serv Deliv Res. 2014;2(21). 25642551

[CR34] Pergolizzi JV, Raffa RB, Tallarida R, Taylor R, Labhsetwar SA (2012). Continuous multimechanistic postoperative analgesia: a rationale for transitioning from intravenous acetaminophen and opioids to oral formulations. Pain Pract.

[CR35] Pillai SB, van Rij AM, Williams S, Thomson IA, Putterill MJ, Greig S (1999). Complexity- and risk-adjusted model for measuring surgical outcome. Br J Surg.

[CR36] Pol RA, van Leeuwen BL, Visser L, Izaks GJ, van den Dungen JJ, Tielliu IFJ, Zeebregts CJ (2011). Standardised frailty indicator as predictor for postoperative delirium after vascular surgery: a prospective cohort study. Eur J Vasc Endovasc Surg.

[CR37] Pomposelli JJ, Gupta SK, Zacharoulis DC, Landa R, Miller A, Nanda R (1997). Surgical complication outcome (SCOUT) score: a new method to evaluate quality of care in vascular surgery. J Vasc Surg.

[CR38] Potgieter R, Hindley H, Mitchell D, McCleary J. Delivering a National Quality Improvement Programme for Patients with Abdominal Aortic Aneurysms. The Vascular Society of Great Britain and Ireland; 2012.

[CR39] Qiu W, Chavarro J, Lazarus R, Rosner B, Ma J (2012). powerSurvEpi: Power and Sample Size Calculation for Survival Analysis of Epidemiological Studies.

[CR40] Rassweiler JJ, Rassweiler M-C, Michel M-S (2012). Classification of complications: is the Clavien-Dindo classification the gold standard?. Eur Urol.

[CR41] Royal College of Physicians of London. National Early Warning Score (NEWS): Standardising the Assessment of Acute-Illness Severity in the NHS. London: Royal College of Physicians; 2012.

[CR42] Sanders J, Patel S, Cooper J, Berryman J, Farrar D, Mythen M, Montgomery HE (2011). Red blood cell storage is associated with length of stay and renal complications after cardiac surgery. Transfusion (Paris).

[CR43] Sanders J, Keogh BE, Van der Meulen J, Browne JP, Treasure T, Mythen MG, Montgomery HE (2012). The development of a postoperative morbidity score to assess total morbidity burden after cardiac surgery. J Clin Epidemiol.

[CR44] Schouten O, Kok NFM, Hoedt MTC, van Laanen JH, Poldermans D (2006). The influence of aneurysm size on perioperative cardiac outcome in elective open infrarenal aortic aneurysm repair. J Vasc Surg.

[CR45] Slankamenac K, Graf R, Barkun J, Puhan MA, Clavien P-A (2013). The comprehensive complication index. Ann Surg.

[CR46] Snowden CP, Prentis JM, Anderson HL, Roberts DR, Randles D, Renton M, Manas DM (2010). Submaximal cardiopulmonary exercise testing predicts complications and hospital length of stay in patients undergoing major elective surgery. Ann Surg.

[CR47] Strasberg SM, Linehan DC, Hawkins WG (2009). The accordion severity grading system of surgical complications. Ann Surg.

[CR48] Streiner DL (2003). Being inconsistent about consistency: when coefficient alpha does and doesn’t matter. J Pers Assess.

[CR49] Visser L, Pol RA, Tielliu IFJ, van den Dungen JJAM, Zeebregts CJ (2014). A limited and customized follow-up seems justified after endovascular abdominal aneurysm repair in octogenarians. J Vasc Surg.

[CR50] Wakeling HG, McFall MR, Jenkins CS, Woods WGA, Miles WFA, Barclay GR, Fleming SC (2005). Intraoperative oesophageal Doppler guided fluid management shortens postoperative hospital stay after major bowel surgery. Br J Anaesth.

[CR51] West MA, Lythgoe D, Barben CP, Noble L, Kemp GJ, Jack S, Grocott MPW (2014). Cardiopulmonary exercise variables are associated with postoperative morbidity after major colonic surgery: a prospective blinded observational study. Br J Anaesth.

